# Headaches of the Decline of Life

**Published:** 1876-07

**Authors:** 


### HEADACHES OF THE DECLINE OF LIFE.

S. Weir Mitchell, M. D., of Philadelphia, contributes the following to the *Medical and Surgical Reporter:* "These cephalagias are for me always full of suspicion. If a person who has been free of headaches begins late in middle life to have them, the case is usually one which will need every care we can give it. In such cases, after excluding the eyes as the cause, it is most needful to make sure that the headache be not remotely due to albuminuria, from contracted kidneys. In an article in the Philadelphia *Medical Times* for August, 1874, on the nervous accidents of albuminuria, I have already spoken of this matter, and have there given three cases of headache, in all of which albuminuria was the unsuspected parent of the pain. But, after putting aside these and the still more common causes of headaches, as gastric disorder and the constipation of old age, there yet remain headaches which have often, I think, some relation to causes which in the old produce hemiplegia. These headaches are apt to occur on one side of the head, or to be most felt on one side when even the whole head aches. They are liable to be attended by a sense of fullness and by throbbing, and they are extremely apt to be felt every morning on awakening from sleep. Headache is one of the near prodromes of hemiplegia, according to the books, but in my experience is not a common one; while, as a more remote warning, it has value, but still not very frequent. I have hesitated in these brief clinical sketches to speculate much on the causes of symptoms, nor do I see my way here to say what it is in the state of a head with degenerating vessels which gives rise to pain; yet, practically speaking, I am sure of the fact. I every now and then meet a man who has headache and slight numbness on one side, and who may or may not have had a slight hemiplegia. I bleed this man by leeches a few ounces. I am perfectly sure he will be free of pain and eased of numbness for some time to come. I take the blood from the temple and from the back of the ear on the worst side. The immediate connection in these regions with the brain-breeding vascular areas beneath them is clear and abundant, and it does seem as if the local depletion eased a local overplus, and that the distended vessels did not give way anew for some time to come; but this is a speculation merely, while the valuable fact as to the use of leeching rests unchanged, however we explain or do not explain it. Hard, too, to fully comprehend is the other fact, as to which I am quite as sure, that in a florid man wall on in the fifties or over them, with a strong heart, throbbing headaches, and hints of hemiplegia in the way of unilateral numbness or tingling, the leech is made of longer use and even of permanent value by restricting the diet to vegetables, milk and fruit. I could easily quote case on case in support of these assertions, but one shall answer: A stout, somewhat ruddy gentleman, aged sixty-one years, from Delaware, called on me two years ago, with the following symptoms: a strong pulse and heart-beat; slightly beaded radial arteries; a faint senile arc; large, tortuous, visibly full temporal arteries; an occasional increasing numbness of the left side, ending in a slight hemiplegia, two years ago, but before this and since he had daily headache on awakening, and of late attacks of dull, throbbing ache, not worse on one side, but when present nearly always accompanied by a sensible over-action of the heart and by increased left-side numbness. Cardiac sedatives and purgatives aided him none, but a full leeching gave immense relief. In three weeks it had to be done again, and in two months yet again; then I urged absolute deprivation of meat, and that has succeeded, so that only once since has he been leeched. Tobacco had something to do with the first of his headaches, and was at least potent in ability to bring one on when used in excess. At last he learned this, and ceased to smoke as much, which presently lessened the number of attacks, but did not prevent them altogether. At last he acquired curious cardiac sensitiveness to tobacco, which grows on old smokers, and he was forced to abandon it. Nevertheless, the headaches remained. There is one most remarkable fact in this history of neuralgic headache (megrim): it is very apt to cease as men grow old, but, also, it is apt to disappear and return no more in those who have had a single hemiplegia attack, however slight. I find in my note-books seven causes of hemiplegia, three right and four left, in which are noted this most interesting peculiarity." —*Louisville Med. News.*

